# A quantitative approach to barriers in access to diagnosis and treatment among people with cancer (Argentina, 2024)

**DOI:** 10.3332/ecancer.2025.2052

**Published:** 2025-12-02

**Authors:** Clara Pierini, Clara Mariano y Jelicich, Fabiola Bascuñán Acuña, María De San Martín, Aldana Casati, Cecilia Casullo, Marta Díaz Madero, Delfina Grennon Viel, Estefania Marzik, Gabriela Rodriguez, Victoria Viel Temperley

**Affiliations:** 1 Donde Quiero Estar Foundation, University of Buenos Aires, Buenos Aires, Argentina; 2 Donde Quiero Estar Foundation, University of Chile, Santiago, Chile; 3 Donde Quiero Estar Foundation, National University of the Arts, Buenos Aires, Argentina; 4 Donde Quiero Estar Foundation, University of Salvador, Buenos Aires, Argentina; 5 Donde Quiero Estar Foundation, Kennedy University, Buenos Aires, Argentina; 6 Donde Quiero Estar Foundation; 7 Donde Quiero Estar Foundation, UADE, Buenos Aires, Argentina; 8 Donde Quiero Estar Foundation, INEF, Buenos Aires, Argentina

**Keywords:** accessibility to health services, cancer patient pathway, barriers to access to health services, cancer diagnosis and treatment

## Abstract

**Introduction:**

The limited information available on the cancer patient pathway in Argentina and barriers to access during diagnosis and treatment created a need to produce updated data that would support possible interventions.

**Objectives:**

To survey and analyse access to cancer diagnosis and treatment from the patient's perspective in Argentina (2024).

**Method:**

A quantitative methodology was used, employing a survey method. Sampling was non-probabilistic. It was decided to work in four jurisdictions and with adults with any type of cancer. Quotas were established by sex, jurisdiction and type of cancer.

**Results:**

Fifty percent of respondents reported difficulties in accessing the first consultation. These limitations were more frequent among patients with colon cancer (63%), users of the public subsector (67%) and residents of the Autonomous City of Buenos Aires (56%). Fifty percent of the sample experienced difficulties in accessing diagnosis, a percentage that would increase among people with lung cancer (57%), those with health insurance coverage (57%) and those residing in Misiones (62%) and Tierra del Fuego (68%). At this stage, the main obstacles were related to a lack of appointments, bureaucratic hurdles and distances to health centers. The latter two barriers also stood out in access to treatment, where 63% of respondents encountered difficulties. Added to these were delays in the delivery of medications. Residents of Buenos Aires and Tierra del Fuego reported greater obstacles at this stage (73% and 67%, respectively). Regarding access to medication, 48% of respondents reported having encountered difficulties. This percentage also varied according to gender, type of cancer, jurisdiction and type of coverage. Finally, the average time between the first consultation and the start of treatment was 130 days for the total sample (SD = 122).

**Discussion:**

Barriers to accessing cancer diagnosis and treatment are a concern, as they can affect the progression of the disease or lead to treatment abandonment. Inequalities in access are observed according to jurisdiction and type of coverage, which would reflect territorial disparities and disparities related to the ability to pay in Argentina.

## Introduction

This article presents the initial findings of a broader study conducted by the Fundación Donde Quiero Estar (FDQE), which aimed to survey and analyse the process of cancer diagnosis and treatment from the patient's perspective in Argentina (2024). The focus here was on characterising access for people with cancer and identifying the main barriers that would present themselves.

This effort seeks to understand the journey of people with cancer and its characteristics, with a view to producing quality, up-to-date and accessible information that would be used (to plan actions) by private, state and civil society organisations. The FDQE's motivation for pursuing this study was that the information on the cancer patient's journey is still scarce and even cancer data records in Argentina and Latin America only provide a partial picture of the cancer problem and how it evolves [1].

It would be worth mentioning that the FDQE is an organisation with almost 20 years of experience working with people with cancer. Although it is based in Buenos Aires, it carries out its activities throughout Argentina. The Foundation carries out various lines of work: it accompanies patients during chemotherapy through art and reflexology; it provides emotional support and personalised assistance; it produces guides on procedures and reliable, centralised information on different types of cancer^1^ and it accompanies people so that they can access their medication and continue with their cancer treatment. In addition, it is the founder and coordinator of the national network Unidos por el Cáncer (United Against Cancer), which would bring together 150 civil society organisations and patient groups in Argentina, and is the founder of the Latin American Initiative for Cancer Control. In 2023, the organisation created a research area that investigates access to healthcare for people with cancer and is in charge of the study ‘Mapping people with cancer’ (MAPEC).

### Theoretical-conceptual framework

Access can be approached from two different perspectives. The more traditional approach focuses on the provision of services, considering their characteristics and the factors that facilitate or would hinder their use by users. While these aspects are decisive for the use of services, they are not always the only ones. It is therefore essential to adopt a people-centered notion of access. Access to the health system can vary even among those who share the same geographical area and similar socioeconomic and cultural conditions. This concept encompasses not only physical distances and means of transportation, but also personal and family factors, the nature of the service sought and the sociohistorical context, among other aspects. In this sense, individuals play an active role as builders of the conditions necessary to access the system. This perspective will be the focus of our study, which aims to identify the barriers that people with cancer would face when approaching the healthcare system. These barriers can be classified as geographical, cultural, administrative and bureaucratic, coverage and legal, symbolic and economic [2–7].

It is worth clarifying that the literature distinguishes several stages in the cancer patient's journey [8,9]. Here, we recognise the following: diagnosis, treatment, follow-up, discharge and palliative care, and we will put attention on access to care and the barriers that arise during the first two stages.

### A brief description of Argentina and its healthcare system

Although the right to health is constitutional in Argentina and the public health subsystem is free and universal, effective access to services is strongly conditioned by the segmented and fragmented structure of the system, which would generate profound inequalities.

The healthcare system is organised into three subsystems – public, social security (social welfare) and private (prepaid medicine) – which operate in parallel, with little coordination between them and with very different operating, financing and coverage logics, meaning that the population is not offered a homogeneous package of services [7]. The high levels of fragmentation and segmentation between subsectors (and within them) result in inequalities in both coverage and access to services (in other words, the care that each person would receive depends largely on their ability to pay or type of coverage) [10–12]. In addition, there is an unequal distribution of resources across the country[13,14].

Finally, the federal nature of the system adds an additional layer of complexity. Argentina has 24 jurisdictions: 23 provinces and 1 federal district, the Autonomous City of Buenos Aires (CABA). Health care in the country falls under the purview of these jurisdictions, which would be responsible for defining the scope, content and organisation of services within their boundaries. These jurisdictions would establish their municipal regimes and define areas of competence at the local level [15,16]. This situation creates a notable tension in terms of ‘rights’ and ‘autonomy,’ since policies aimed at universality are implemented in health care providers outside the sphere of the nation [17].

In this article, as explained in the following section, we will work with four jurisdictions: the CABA and the provinces of Buenos Aires, Misiones and Tierra del Fuego. As shown in Table 1, the selected jurisdictions have markedly dissimilar characteristics, both in socioeconomic terms and in the organisation and capacity of their health systems.

## Methods

This article is part of a broader study that would have used a quantitative methodology and employed the survey method. The research team defined a series of variables to be surveyed, in line with its objectives. These variables were operationalised into questions through a questionnaire. Table 2 presents the variables used in this article.

The main data collection technique was an *ad hoc* questionnaire consisting mainly of closed or semi-closed questions, although some open questions were included. The questionnaire addressed the following dimensions: a) Sociodemographic characteristics of the patient, b) Start of the cancer patient's journey, c) Diagnosis, d) Medication and treatment and e) Support during the patient's journey. It consisted of 56 questions.

The sampling was non-probabilistic and the sample was not representative. Thus, the sample does not represent the total population, although it provides an approximation of the situation of cancer patients. The people to be surveyed were accessed in three ways: 1) because they were in contact with the FDQE and are part of its databases; 2) because they are treated at health facilities where the FDQE is present and the health team treating them would offer them the opportunity to participate in the study and they agreed and 3) because they signed up as volunteers to participate through the FDQE's social media, where awareness campaigns were carried out.

The units of analysis were people with any type of cancer, over the age of 18, who had accessed diagnosis and treatment at a healthcare facility located in the City of Buenos Aires or in the provinces of Buenos Aires, Misiones or Tierra del Fuego during the last 5 years. Only patients who would have started treatment at least 6 months ago were included.

It was decided to work with people with any type of cancer, given that the FDQE would not limit its work to a specific type and was interested in obtaining general information about the cancer patient's journey. Given the high incidence of breast cancer, lung cancer and colon cancer worldwide and in Argentina [1, 8], specific quotas were established for these types within the sample (at least 20 cases). The choice of jurisdictions was based on the following reasons:

The City of Buenos Aires and the Province of Buenos Aires are the jurisdictions with the largest populations in Argentina and also have a large number of establishments in the three subsectors that would make up the health system.

- Misiones is among the jurisdictions with the highest age-adjusted mortality rate from cancer in Argentina [18].
- The Province of Tierra del Fuego was selected as representative of the Patagonian region and because it is not among the jurisdictions with the highest age-adjusted mortality rate from cancer in Argentina (it ranks eleventh among the 24 jurisdictions).

In addition, in the selected jurisdictions, the FDQE has extensive experience working with and contact with health facilities and civil society organisations that facilitated access to the field.

The decision to survey people who had been diagnosed and started treatment in the last 5 years was made so that respondents could easily remember dates, places, decisions and other characteristics of their cancer journey. Individuals had to have started treatment at least 6 months prior to the survey date to ensure they had experience within the healthcare system and were able to share sensitive issues (when the diagnosis is recent, people are usually very emotional).

The questionnaire was laid out on the *Jotform* online platform and designed to be completed by telephone. It was administered by social scientists who work at the FDQE or would be hired by it. Before going into the field, the team of interviewers was trained by the researchers in charge of the study.

A total of 153 surveys were conducted as part of the study. Table 3 summarises the characteristics of the sample. As can be seen in the table, the jurisdictions where individuals received both diagnosis and treatment were considered, given that in some cases these processes would not take place in the same jurisdiction. In terms of the types of cancer that participants are experiencing or have experienced, the distribution was as follows: 61 cases of breast cancer, 27 of colon cancer, 21 of lung cancer and 44 of other types. Regarding the health coverage of the people surveyed, 63 cases had social security, 57 had private or mutual health plans, 30 had exclusive public health coverage and 3 cases did not know how to answer.

The information obtained during the *Jotform* surveys was recorded in *Salesforce software*. The data were managed there, since uploading the surveys would generate a database that includes the answers to the different questions asked of each respondent. This platform was used to monitor the fieldwork, taking into account the planned quotas. For processing and analysis, the database was exported to *SPSS software*.

Before conducting the survey, the purpose of the study and the voluntary nature of their contribution were explained to each participant. They were then asked for their informed consent. The data used were anonymised to preserve privacy and confidentiality, in accordance with Law No. 25326 on the Protection of Personal Data.

This study did not require review by an Ethics Committee, as it falls within the exceptions to review by an Ethics Committee on Research (specifically: study of health systems). The exceptions are defined by the Guide for Research on Human Health approved by Resolution 1480/2011 of the Ministry of Health of the Nation.

## Results

### Start of the patient's journey (first consultation)

When asked about their first consultation related to the disease, 50% of respondents reported having faced difficulties in accessing it. These proportions remained constant between men and women. However, when analysed by type of cancer, patients with colon cancer reported greater difficulties (63%), followed by those with breast cancer and other types of cancer (48%) and finally, patients with lung cancer (42%). In terms of coverage, 67% of patients treated in the public subsector faced obstacles at this stage, 50% of those with health insurance and 39% of patients in the private subsector also reported problems. At the jurisdictional level, Misiones recorded the lowest proportion of obstacles (38%) to accessing the first consultation, while in Tierra del Fuego (48%) and Buenos Aires (50%), the results were consistent with the overall average. In contrast, in the CABA, 56% of patients encountered difficulties. The dismissal of symptoms appeared to be the main barrier at this stage in 30% of cases. This figure reached 50% among patients with colon cancer. Other difficulties identified are related to insufficient appointments (27%) and excessive delays in assigning appointments (26%).

### Diagnosis

When referring to the diagnosis stage, around 50% of those surveyed indicated that they would have difficulties accessing it. This percentage varies slightly when we analyse access difficulties by gender (42% among men and 53% among women). When we consider the type of cancer, some variations appear: 51% among breast cancer patients, 57% among lung cancer patients, 44% in cases of colon cancer and 52% among those with other types of cancer. When looking at the results by type of coverage, the percentages would also be close to 50 (42% among those affiliated with private or mutual plans, 50% for people treated in the public subsector and 57% for users of social welfare programs). The analysis of the results by jurisdiction shows some variations, as shown in Table 4.

The questionnaire asked about the type of difficulties encountered in relation to access to diagnosis. Among the list of possible answers, only four were selected by those who encountered obstacles: bureaucratic or coverage difficulties (24%), difficulties in getting appointments (22%), appointments given with long delays (21%) and distance from home to health facilities (18%). The rest of the options reached percentages of less than 10%.

Among the obstacles encountered, some appeared more frequently in certain groups of patients. For example, in the case of people with colon cancer, 50% mentioned having difficulties related to appointments (difficulty in obtaining them or long delays) and 25% of people with lung cancer indicated difficulties in reaching a diagnosis (for example, their lesions were difficult to biopsy). Bureaucratic difficulties reached higher percentages among those diagnosed in Misiones (44%) and Tierra del Fuego (31%).

### Treatment

Sixty-three percent of respondents had difficulties accessing treatment. This proportion remains stable among women (61%) and reaches 73% among men. This figure decreases in the cases of patients who underwent treatment in CABA (53%) and Misiones (52%) and increases in the provinces of Buenos Aires (73%) and Tierra del Fuego (67%). It is noteworthy that 20% of those who were diagnosed in Tierra del Fuego would undergo treatment in other jurisdictions. Eight percent of respondents were diagnosed in one jurisdiction and migrated to another for treatment.

Among those who identified barriers to accessing treatment, 39% highlighted delays in the delivery of medications. This percentage is even higher among patients in CABA (44%) and Buenos Aires (46%), and among people with colon cancer (50%). Those with exclusive public coverage mentioned this difficulty in 56% of cases. Bureaucratic difficulties appeared as the second obstacle to accessing treatment in 31% of cases. Among lung cancer patients and those with private health insurance, it appeared in 44% of cases. The distance from home to health centers (18%) and difficulties in traveling to them (13%) appeared as the third and fourth barriers to accessing treatment.

Forty-eight percent of respondents reported difficulties in accessing medication. This proportion remained similar among women (46%), but increased among men (62%). In terms of cancer types, breast cancer had the lowest percentage (39%), while lung cancer (52%), colon cancer (56%) and other types (55%) exceeded the overall average. At the jurisdictional level, a lower proportion of those who underwent treatment in Misiones faced obstacles in accessing medication (37%), followed by those treated in CABA (40%), Tierra del Fuego (53%) and Buenos Aires (59%). When analysing difficulties according to health coverage, 35% of private or mutual plan users encountered obstacles in accessing medication. This figure rises to 52% among those with social security and 63% among users of exclusive public coverage. Among the main barriers identified, medication delivery times were the most frequent, affecting 58% of cases, followed, albeit at a considerable distance, by coverage authorisation times, in 39% of cases.

### Time elapsed between the first consultation and the start of treatment

This study collected data on the dates of the first consultation related to the disease and the start of treatment, which would allow calculating the average delay between the two events. Overall, the average delay was 130 days, with a standard deviation of 122 days, indicating high variability in the delays between the cases analysed. In the case of men, this time decreases to 90 days and among women, it increases to 138 days. Among the different types of cancer, those who underwent treatment for breast cancer averaged 125 days between the first consultation and the start of treatment, in cases of colon cancer, 117 days, lung cancer, 178 days and 120 days for other types of cancer. The average number of days also varies according to coverage: patients with private health insurance or mutual insurance 104, with exclusive public coverage 130 and those affiliated with social welfare programs 153. Table 6 details the average time between the first consultation and the start of treatment according to the jurisdiction of diagnosis.

Among those who did not encounter difficulties in obtaining medication, the average time between the first consultation and the start of treatment was 110 days. In contrast, for those who did encounter obstacles, this average increased to 151 days. Specifically, among those who had problems with medication delivery times, the average rose to 159 days, while for those who faced delays in coverage authorisation, the average was even higher, reaching 187 days.

## Discussion

We would know that one of the fundamental problems associated with cancer control is the inefficient distribution of resources. This situation can be explained by three major problems that would characterise Latin America: fragmentation within health systems; inequality in service provision based on purchasing power and geographical differences in service provision [1, 9]. Consequently, this analysis considers results disaggregated by jurisdiction, type of coverage and type of cancer.

If we analyse the percentage of people who would report difficulties in accessing diagnosis by jurisdiction, the differences between CABA (39%) and Tierra del Fuego (68%) are cause for concern. Tierra del Fuego also has a high percentage of people who encountered difficulties in accessing treatment (67%) and is the province with the highest number of days between the date of the first consultation related to the disease and the start of treatment (170) among those who were diagnosed there. It is surprising that 20% of patients who were diagnosed in that province continued their treatment in another jurisdiction. On the other hand, when analysing the percentage of the sample that faces difficulties in accessing medication, it can be seen that in some jurisdictions, such as Buenos Aires (59%) and Tierra del Fuego (51%), the values exceed the average. This situation highlights the dissimilar scenarios in relation to access to oncological care that exist between the jurisdictions of Argentina.

When considering the data related to coverage, differences between users of different subsectors are evident. The private subsector shows the lowest percentages of patients who would encounter difficulties in accessing the first consultation (39%), diagnosis (42%) and treatment (49%). In addition, it has the lowest average number of days between the first consultation related to the disease and the start of treatment (104) and the lowest proportion of difficulties in accessing medication. For its part, although the public subsector is within the average in terms of difficulties in accessing diagnosis and the time to start treatment, it shows a high proportion of patients with difficulties in accessing the first consultation (67%), treatment (87%) and medication (63%). In the case of social welfare programs, the time to start treatment is above average (153 days), as are difficulties in accessing diagnosis (57%), while access to the first consultation is within the average range. It can be said that the results presented indicate that the path for patients affiliated with private health plans presents fewer barriers or obstacles than would be the case for those with public or health insurance coverage.

Analysis of the results by type of cancer reveals that people with colon cancer would face above-average difficulties, especially due to the dismissal of symptoms at the first consultation, delays in obtaining appointments and difficulties in accessing medication. Lung cancer patients, on the other hand, experience a significantly longer average time between the first consultation and the start of treatment (178 days), which could be related to barriers to accessing diagnosis. In contrast, breast cancer patients have relatively favourable access compared to other types of cancer.

The emergence of barriers when accessing the first consultation, diagnosis or treatment often directly affects the prolongation of the disease or even leads to treatment abandonment. The obstacles that arise in the path of people with cancer confront them with the need to implement strategies and also to have resources to overcome them (e.g., time and money). Furthermore, the time would takes to complete the process from the first consultation related to the disease to the start of treatment is a variable of utmost importance for the progression of the disease and the clinical outcome of the patient [20, 21]. Analysis by jurisdiction and type of coverage reveals inequalities in access to cancer care throughout Argentina and according to patients' ability to pay.

The results obtained reveal that people with cancer would face significant barriers throughout their journey with the disease. Among the most frequent are the dismissal of symptoms during the first consultation, bureaucratic difficulties, coverage problems, limitations in obtaining and availability of appointments, the distance between home and health centers and complications in accessing medication. This last obstacle, in particular, causes a delay in the start of treatment: people who face problems obtaining medication begin their treatment, on average, 41 days later than those who would not experience this difficulty. Furthermore, the analysis shows that those who would experience bureaucratic barriers (especially problems related to authorisations) begin their treatment 57 days later than the average. The findings show that barriers to accessing cancer care during the early stages (first consultation, diagnosis and treatment) are closely linked to bureaucratic, coverage and geographic factors.

It is worth noting that these barriers appear in other studies that would investigate access to healthcare for people with cancer. For example, a Brazilian study refers to the problems experienced throughout the care process by lung cancer patients and their families who are treated in the public sector, indicating: difficulty in accessing medication and tests, queues and long waits to receive care, slow diagnosis, among other issues [8].

## Relevance for health policies and interventions

The article offers results that could be used as feedback for designing healthcare systems. Patient experience and satisfaction are important aspects when evaluating the quality of care, facilitating access to health services and positively influencing survival rates. Highlighting the barriers that most frequently arise among people who would have undergone cancer treatment will help to identify improvements in health policies related to this disease. Inequalities between jurisdictions and types of coverage indicate the need to strengthen resources and infrastructure in certain cases, as well as the need for resource redistribution.

Specifically, based on the results of the study, the Foundation will promote two lines of action aimed at transforming the evidence into concrete improvements. On the one hand, patient training and guidance programs will be strengthened with new content based on MAPEC's findings. The goal is to promote informed decisions and more equitable access to cancer care.

On the other hand, it proposes the creation of a multisectoral working group to collaboratively address the barriers identified. This initiative will consist of a series of face-to-face meetings with key players in the cancer ecosystem (government officials, hospitals, chambers of commerce, scientific societies, civil society organisations, among others) in order to: validate and enrich the study's results, identify structural causes for the problems identified and opportunities for innovation, formulate recommendations and articulate commitments for their implementation. The process will conclude with the signing of a ‘Pact for the Future.’

## Conflicts of interest

There was no conflicts of interest during the conduct of the study.

## Funding

Donde Quiero Estar Foundation and six laboratories (Pfizer, Takeda, MSD, Novartis, Roche, and AstraZeneca).

## Author contributions

All authors have made a substantial contribution to the conception or design of the study or to the collection, analysis or interpretation of the data; have participated in the writing of the article or in the critical review of its intellectual content; have approved the final version of the manuscript and are able to respond to all aspects of the manuscript to ensure that issues related to the accuracy or integrity of all its contents have been adequately investigated and resolved.

## Figures and Tables

**Figure 1.**
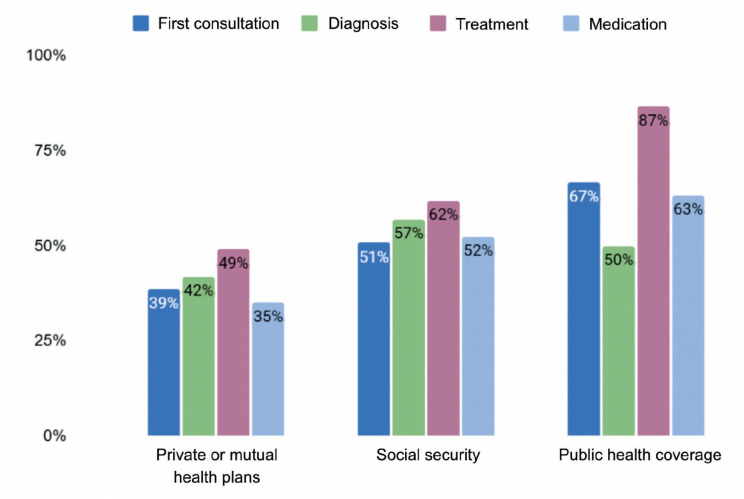
Difficulties in accessing different stages of the patient pathway by type of coverage, in percentage values.

**Figure 2.**
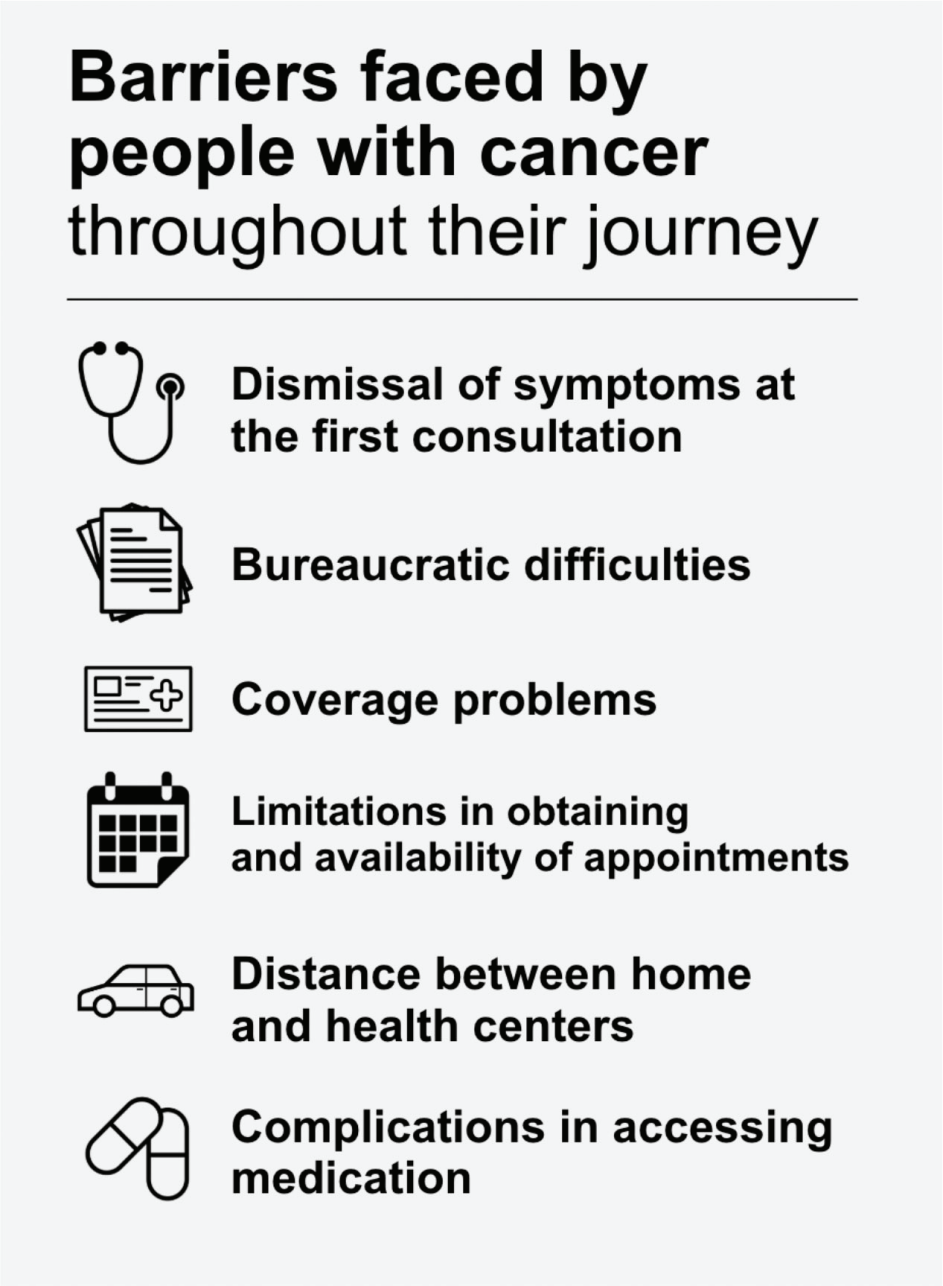
Barriers faced by people with cancer throughout their journey with the disease.

**Table 1.** Characteristics of the selected jurisdictions.

	Population size*	Population density (inhabitants/km^2^)*	Population with exclusive public coverage (%)*	Total number of beds (*N*)***	Health facilities (*N*)**	Physicians (*N*)****
Country total	45,892,285	12.5	36	168,293	33,684	186,137
Buenos Aires	17,523,996	57.3	35	65,141	8,553	49,429
CABA	3,121,707	15,161.3	16	23,005	1,291	52,907
Misiones	1,278,873	42.8	47	3,805	930	2,509
Tierra del Fuego	185,732	0.2	15	446	461	886

Sources:

* Population size, population density and coverage. National Population, Household and Housing Census (INDEC), 2022

** Total number of healthcare facilities. All subsectors. Federal Registry of Healthcare Facilities (REFES), 2022

*** Total number of beds available (excluding beds for the elderly). REFES, 2022

**** Number of physicians. Federal Observatory of Human Talent in Health (OFETHUS), based on the Federal Network of Health Professional Registries (REFEPS), 2022

**Table 2.** Variables considered for this article.

Variables considered	
Gender	Date of first consultation related to the disease
Date of birth	Difficulties encountered in accessing the first consultation
Age	Jurisdiction of diagnostic procedure
Place of residence at the time of diagnosis	Difficulties encountered in accessing diagnosis
Place of residence at the start of treatment	Difficulties associated with obtaining medication
Current main occupation	Date of initiation of treatment pharmacological
Health coverage	Place where treatment is carried out
Type of cancer	Difficulties encountered in accessing treatment

**Table 3.** Characteristics of the sample. Gender and jurisdiction of diagnosis and treatment of participants.

		Jurisdiction of diagnosis					Total
		Buenos Aires	CABA	Tierra del Fuego	Misiones	Others	
Gender	Male	9	12	3	2	0	26
	Women	57	29	16	24	1	127
Total		66	41	19	26	1	153
		Treatment jurisdiction					
Gender	Male	9	12	3	2	0	26
	Women	54	35	12	25	1	127
Total		63	47	15	27	1	153

**Table 4.** Percentage of people who reported difficulties in accessing diagnosis by jurisdiction where diagnosis was made.

	% of people who would indicated difficulties in accessing diagnosis
CABA	39%
PBA	48%
Misiones	62%
Tierra del Fuego	68%

**Table 5.** Percentage of respondents who would have difficulty accessing treatment by type of cancer and health coverage.

Existence of difficulties in accessing treatment	Type of cancer				Type of coverage		
	Breast	Colon	Lung	Other types	Private health insurance or mutual insurance	Social welfare	Public coverage
Had difficulties	54%	74%	76%	61	49%	62%	87%

**Table 6.** Days between the date of the first consultation related to the disease and the start of treatment according to jurisdiction of diagnosis.

Jurisdiction of diagnosis	Time elapsed until treatment in days
CABA	129
Buenos Aires	130
Misiones	100
Tierra del Fuego	173

## Footnotes

1 Available at https://mundocancer.org/

## References

[1] National Cancer Institute, Ministry of Health of the Nation (2024). Incidence.

[2] Gutiérrez A (2011). Inputs for intersectoral public policy management: mobility and access. Territorios.

[3] Frenk J (2014). The concept and measurement of accessibility. Salud Públ México.

[4] Ballesteros M (2014). An Analysis of Inequalities in Access to Health Services Among the Urban Adult Population of Argentina Based on Secondary Data. Documents from Young Researchers.

[5] Comes Y, Solitario R, Garbus P (2007). The concept of accessibility: the relational perspective between population and services 14.

[6] Maceira D (2009). Inequality in Access to Health Care in Argentina.

[7] Mota RT, Martins EF, Vieira MA (2021). Care pathway for patients living with lung cancer. Rev Bioética.

[8] Rubio AR, Mareque M, Soto J (2022). The patient experience in lung and breast cancer through the patient journey. Farm Hospitalaria.

[9] Acuña CH, Chudnosky M (2002). The Health System in Argentina.

[10] Belmartino S (2005). Argentine Healthcare in the 20th Century. Institutions and processes.

[11] Cetrángolo O (2014). Fragmented financing, unequal coverage, and lack of equity in the Argentine health system. Buenos Aires J Polit Econ.

[12] Abramzón MC (2006). Human resources in health in Argentina. An ongoing challenge. J Public Health.

[13] Maceira D, Olaviaga S, Kremer P (2006). Primary Health Care Centers: an overview of their distribution in Argentina.

[14] Damsky IA (2006). The construction of the right to health in Argentina based on the Internationalization of legal systems.

[15] Abramovich V, Pautassi L (2009). The rights-based approach and the institutional framework of social policies. Judicial review of social policies: case studies.

[16] Chiara M (2013). Health Care Policy and Intergovernmental Relations (RIG): Continuities and Inflections in Patterns of Interaction in Greater Buenos Aires, Argentina (2001–2011) [doctoral thesis].

[17] National Cancer Institute, Ministry of Health of Argentina (2024). Mortality.

[18] Kielstra P, Koehring M (2017). Cancer Control, Access, and Inequality in Latin America: A Story of Light and Shadow.

[19] Torrente Salazar S (2020). Barriers to Access to Health Services in Breast Cancer Care in the City of Bogotá DC.

[20] Arrossi S, Curotto M, Zalacaín Colombo J (2019). Cervical Cancer Prevention: Protocol for Implementing the Navigators Strategy in a Programmatic Context.

[21] Tobar F (2002). Access to Medicines in Argentina: Diagnoses and Alternatives. Health and Public Policy Seminar Series.

